# Exploring robotic total hysterectomies: a multi-site experience with the Senhance Surgical System

**DOI:** 10.1007/s11701-024-01944-4

**Published:** 2024-06-26

**Authors:** Burghard Abendstein, Michael Prugger, Attila Rab, Raimondas Siaulys, Vaida Nausediene, Rita Karpiciute, Frank Willeke, Narimantas Evaldas Samalavicius

**Affiliations:** 1 Department of Gynaecology, Academic Teaching Hospital Feldkirch, Carinagasse 47, 6800 Feldkirch, Austria; 2 Department of Gynaecology, Klaipeda University Hospital, Klaipeda, Lithuania; 3 Clinic of Abdominal and Thoracic Surgery, Klaipeda University Hospital, Klaipeda, Lithuania; 4 Faculty of Health Sciences, Management of Human Health Activities, Klaipeda University, Klaipeda, Lithuania; 5 Department of Day Surgery, National Cancer Institute, Santariskiu 1, Vilnius, Lithuania; 6 Department of General and Visceral Surgery, Marien Hospital, Siegen, Germany; 7 Faculty of Medicine, Institute of Clinical Medicine, Vilnius University, Vilnius, Lithuania; 8 Health Research and Innovation Science Center, Faculty of Health Sciences, Klaipeda University, Klaipeda, Lithuania

**Keywords:** Robotic surgery, Total hysterectomy, Senhance surgical system, Multi-site experience

## Abstract

Robotic-assisted surgery emerged as a technological advancement in the twentieth century, with gynaecology being a key adopter of this approach. The Senhance Surgical System has gained prominence for total hysterectomies from single-site experiences, but multi-site reporting are still lacking in present literature. This multi-site study, conducted at Klaipeda University Hospital and Academic Teaching Hospital Feldkirch, aimed to explore the safety and feasibility of total hysterectomies with the Senhance Surgical System. The study involved 295 cases, showcasing a well-established routine with minimal procedure times. The average age of the patients was 53.5 years (SD: 10.3 years), ranging from 18 to 80 years. The patients’ BMI averaged 25.6 kg/m^2^ (SD: 6.2 kg/m^2^), ranging from a minimum of 17.7 kg/m^2^ to a maximum of 69.5 kg/m^2^. The duration of surgery varied between 30 and 215 min, with a median of 95 min (IQR: 81–116). The docking time was a median of 3 (IQR: 2–5) min and varied between 1.0 and 30.0 min, with a minimum to a maximum range of 1.0 to 122 min. Conversion (3 cases, 1%) and adverse events (6 cases, 2%) were infrequent. Additionally, robotic malfunctions were recorded minimally in 4,1% (12 cases) of the procedures, and pain on a 0–10 visual pain scale was reduced from mild [2.7 (± 1.2)] one day postoperative to minimal [0.9 (± 0.5)] at discharge. Overall, a great routine with the Senhance Surgical System proves good control and, thus, feasibility and safety. Therefore, the Senhance Surgical System is a viable option for total hysterectomy.

## Introduction

Robotic-assisted surgery represents a notable technological advancement of the twentieth century. While its approach shares similarities with laparoscopic surgery, it offers significant patient advantages, including reduced pain, shorter hospitalisation times, and comparable outcomes with a similar risk of adverse events [1]. Among many specialities, gynaecology has already embraced the approach for various applications. Consequently, gynaecology is a surgical speciality where robotic surgery is one of the most commonly applied methods [2]. Given the systems’ precision, augmentation, and dexterity compared to standard laparoscopy or open surgery, it is reasonable that benign and malignant gynaecological diseases are experiencing rising treatment with robotic systems [3]. A total hysterectomy (TH) is a key procedure in gynaecology. Especially for non-cancerous conditions, the American College of Obstetricians and Gynaecologists Committee on Gynecologic Practice and The Society of Gynecologic Surgeons [4] approved the advantages of robotic-assisted surgery. Still, it encourages surgeons to decide each case based on individual risks [4]. The first robotic experiences with TH were reported with the Da Vinci System (Intuitive Surgical, Inc., Sunnyvale, CA, USA) in 2002 [5]. Several emerging robotic systems are challenging the market dominance of the Da Vinci System (Intuitive Surgical, Inc., Sunnyvale, CA, USA). One of the newer systems is the Senhance Surgical System (Asensus Surgical US, Inc., Durham, NC, USA), which entered the market in 2014 and is now CE-certified and Food and Drug Administration (FDA)-approved for TH. The Senhance Surgical System provides various benefits similar to and even beyond other surgical systems. First, the ergonomically seated surgeon controls via an open console robotic arms independently placed. In this setting, communication with the rest of the team is secured, enhancing safety for both the team and the patient. Secondly, trocar placements are in typical laparoscopic positions for efficacy, enabling a hybrid intervention or rapid conversion if necessary. Third, an eye-tracking system and 3D visualisation allow the surgeon to control the view intuitively. This fully contrasts laparoscopic surgery, where a surgical assistant controls the camera. Additionally, haptic feedback contributes to overall safety since true forces between tissue and instruments (e.g., clip applicators, graspers, scissors) and, thus, e.g., the strength of sutures can be perceived, appraised, and analysed. Lastly, and most importantly, the system provides reusable and sterilisable instruments that add an economic advantage over alternative robotic platforms. A recent publication [6] evaluated this benefit carefully for TH. In their comparison between the Senhance Surgical System and the Da Vinci System, the former achieved lower median instrument costs ($559 vs. $1393, *p*-value < 0.001). Additionally, the robotic surgery with the Senhance Surgical System and the laparoscopic approach did not present a significant difference, indicating similar costs ($559 vs $498, *p*-value = 0.336). Early experiences with the Senhance Surgical System for TH have been published, demonstrating feasibility and safety [7, 8]. In 2019, Rumolo et al. [9] published a review of robotic TH with the Senhance Surgical System, stating the potential for robotic-assisted surgery as a standard procedure in gynaecology even if some aspects, such as operative time and docking procedures, should be investigated further. More recent publications also include the feasibility of TH in obese patients [10]. Even an insightful, direct comparison to the Da Vinci System regarding console time was published [6], presenting insignificant time differences (*p*-value = 0.898). The general results seem promising, but further investigation is still necessary to indicate safety, feasibility and advantages, especially compared to other minimally invasive approaches such as standard laparoscopy or open surgery. As stated, multiple publications have been published, including our own [11, 12]. Still, multi-site experiences are missing. Therefore, the present study aims to explore the safety and feasibility of robotically assisted TH with the Senhance Surgical System at two European sites.

## Methods

### Patients

Patients were recruited and screened for robotic TH at Klaipeda University Hospital in Klaipeda, Lithuania and the Academic Teaching Hospital Feldkirch, Feldkirch, Austria. Both of these sites are part of “The TransEnterix European Patient Registry for Robotic-assisted Laparoscopic Procedures in Urology, Abdominal Surgery, Thoracic and Gynecologic Surgery” (“TRUST”) study group and data are excerpts from this study. Adult patients were included when the indication for TH (benign or malign nature) and no absolute contraindications for laparoscopic surgery (e.g., major cardiac or pulmonary diseases that limit CO_2_ infiltration) were applied. Patients eligible for laparoscopic surgery were offered robotic surgery if suitable. Exclusion criteria were the inability to give informed consent (e.g., cognitive impairment such as dementia), any contraindication for a laparoscopic surgical procedure and life-threatening diseases, limiting the participant’s life expectation to less than 12 months. Intraoperative complications were defined as adverse events with visceral or vascular injury, cardiopulmonary event, major blood loss (more than 500 mL), or blood transfusion. Postoperative complications were defined as adverse events such as infection, including wound infection and dehiscence, sepsis, ileus, bowel obstruction, blood transfusion, and venous thromboembolic events, occurring within 30 days after surgery. The adverse events were considered severe if readmission or repeated surgery was necessary or death occurred. If any complication occurred, the relation to the robotic procedure was defined, and an international safety committee re-evaluated the connection to the robotic procedure. Written confirmed consent to the robotic procedure was obtained and protocolled from each patient. Demographic data was collected before the procedure. Data was pseudonymised and secured in a database.

### Procedure

Robotic TH (with or without salpingo-oophorectomy and with or without pelvic lymphonodectomy) with the support of the Senhance Surgical System was performed via the principles of a standard laparoscopic TH, details, especially regarding trocars and their placement, can be found in a recent paper [12]. In Feldkirch, ICG—Sentinel Lymphadenectomy was routinely performed using 2D-imaging.

The robotic surgical team performing surgery consisted of one surgeon and four assistants (four distinct constellations) in Klaipeda. In Feldkirch, the surgical team consisted of two surgeons and six assistants, which resulted in seven different surgical constellations. For each surgery, “duration of surgery” (in min) and “docking time “ (in min) were documented and defined as follows: “duration of surgery” was defined as time of skin incision to end of suture, and “docking time” was defined of start and end of docking the robot to the trocars. Robotic malfunctions indicate that the robotic system stopped the surgery, while robotic limitations refer to events that did not necessitate stopping the surgery but interfered with the process. When it occurred, conversions to standard laparoscopy, open surgery, and adverse events referring to intraoperative and postoperative were protocolled. Additionally, postoperative and discharge pain scores were documented on a 0–10 visual pain scale.

Study Initiation started in August 2019 in Klaipeda and in January 2022 in Feldkirch. The data extraction from the registry databases ended in November 2023.

Statistical evaluation was performed with “statistical software SAS® 9.4 (TS1M6) for Microsoft Windows” [13]. Data were checked for normal distribution, and adequate statistical evaluation (Mann–Whitney U-Test or Chi-squared Test) were used to evaluate statistical differences. A *p*-value ≤ 0.05 was considered statistically significant. Missing values were not replaced.

## Results

### Demographics

Our sites performed 295 robotic TH, with 236 (80%) undergoing the procedure at the Klaipeda site and 59 (20%) at the Feldkirch site. The average age of the patients was 53.5 years (Standard Deviation (SD): 10.3 years), ranging from 18 to 80 years. The patients' BMI averaged 25.6 kg/m^2^ (SD: 6.2 kg/m^2^). The larger part of the study population did not present a history of smoking (78.0%), relevant diseases (84,7%), or a history of previous abdominal surgery (87.8%). Three patients identified themselves as transgender and thus as male. For a comprehensive overview of demographic data, see Table 1.

**Table 1:** Demographic data and medical history

	N	Klaipeda	Feldkirch	Total
		236	59	295
Age (years)	Mean ± SD	54.4 ± 9.6	49.9 ± 12.1	53.5 ± 10.3
Gender	Female	236 (100%)	59 (100%)	295 (100%)
BMI (kg/m^2^)	Mean ± SD	25.5 ± 6.5	26.3 ± 5.1	25.6 ± 6.2
History of smoking	No	187 (79.2%)	43 (72.9%)	230 (78.0%)
	Yes	49 (20.8%)	16 (27.1%)	65 (22.0%)
Relevant diseases	No	213 (90.3%)	37 (62.7%)	250 (84.7%)
	Yes	23 (9.7%)	22 (37.3%)	45 (15.3%)
Type of relevant diseases (multiple entries)	Diabetes	4 (1.7%)	1 (1.7%)	5 (1.7%)
	Hypertension	3 (1.3%)	1 (1.7%)	4 (1.4%)
	Cardiovascular Co-morbidity	–	5 (8.5%)	5 (1.7%)
	COPD or Impaired Respiratory Function	–	3 (5.1%)	3 (1.0%)
	Liver disease	–	1 (1.7%)	1 (0.3%)
	Stroke	1 (0.4%)	1 (1.7%)	2 (0.7%)
	Sleep apnoea	–	–	–
	GERD	–	1 (1.7%)	1 (0.3%)
	Depression	2 (0.8%)	5 (8.5%)	7 (2.4%)
	Chronic pain	1 (0.4%)	–	1 (0.3%)
	Others	15 (6.4%)	11 (18.6%)	26 (8.8%)
Previous relevant abdominal surgery	No	225 (95.3%)	34 (57.6%)	259 (87.8%)
	Yes	11 (4.7%)	25 (42.4%)	36 (12.2%)
Type of relevant abdominal surgery (multiple entries)	Open surgery	4 (1.7%)	13 (22.0%)	17 (5.8%)
	Laparoscopic surgery	7 (3.0%)	15 (25.4%)	22 (7.5%)

### Procedure time

Based on 295 robotic TH performed with the Senhance Surgical System at both our sites, the duration of surgery varied between 30.0 min and 215 min, with a median of 95.0 min (interquartile range (IQR): 81–116). To illustrate this time further, console time, representing the time the main surgeon actively controls the robotic system, was at a median of 68.0 min (IQR: 50–82) in Feldkirch. The docking time was reported with a median of 3 (IQR: 2–5) min and varying between 1 and 30 min. Duration of surgery did not differ significantly between sites (*p*-value = 0.148, see Table 2). However, docking time results were reported as significantly different (*p*-value = 0.029) between both sites.

**Table 2:** Procedure times. All values are stated in minutes

	N	Klaipeda	Feldkirch	Total	*p*-value^a^
		236	59	295	
Duration of surgery	Median	95	92	95	0.148
	P25%-P75%	85–118.5	79–115	81–116	
Docking time	Median	3	4	3	**0.029**
	P25%-P75%	2–5	3–4	2–5	

Bold value is meant to underline statistical significance

^a^Mann–Whitney U test

### Conversions

All procedures were finished successfully. However, conversions indicating an abandoned usage of the Senhance Surgical System in favour of standard laparoscopic and/or open surgery were necessary in a minority of cases (1%, 3 cases). Two cases (0,7%, one case at each site) needed continuation via open surgery. One case was first attempted with a laparoscopic approach and was then converted to open surgery (see Table 3). In most cases, the reasons for conversions were technical limitations due to difficult (pelvic) anatomy. Only one case needed conversion based on an adverse event.

**Table 3:** Conversion, malfunctions and limitations

	N	Klaipeda	Feldkirch	Total	*p*-value^a^
		236	59	295	
Conversion	No	235 (99.6%)	57 (96.6%)	292 (99.0%)	
	Open	1 (0.4%)	1 (1.7%)	2 (0.7%)	0.076
	lap + open	–	1 (1.7%)	1 (0.3%)	
Robot malfunctions	No	228 (96.6%)	56 (96.6%)	285 (96.6%)	> 0.999
	Yes	8 (3.4%)	2 (3.4%)	10 (3.4%)	> 0.999
Type of robot malfunction (multiple entries)^b^	Console malfunction	6 (2.5%)	–	6 (2.0%)	0.216
	Monitor/camera malfunction	2 (0.8%)	–	2 (0.7%)	0.478
	Other malfunction	2 (0.8%)	2 (3.4%)	4 (1.4%)	0.131
Robotic limitations	Limited motion	74 (31.4%)	1 ( 1.7%)	75 (25.4%)	** < 0.001**
	Collision	23 ( 9.7%)	–	23 ( 7.8%)	**0.013**

Bold values are meant to underline statistical significance

*Open* open surgery, *lap* laparoscopic surgery

^a^ Chi-squared test

^b^two cases at each site involved both ‘Console malfunction’ and ‘Monitor/camera malfunction’

### Limitations and malfunctions

Of 295 robotic TH, robotic malfunctions were documented in a minority of 3.4% of the cases. In detail, most malfunctions were attributed to the console, monitor, or camera. Regarding limitations of the robotic system, limited motion and collisions were recorded in 25.4% (75 cases) and 7.8% (23 cases) of the procedures. A significant site difference was found since Klaipeda reported most of the limited motion and collision. Detailed results are presented in Table 3.

### Adverse events

Six intra- and postoperative adverse events (2%) were documented, with three occurrences at each study site. Four incidents (one in Klaipeda and three in Feldkirch) were recorded before discharge, and two incidents (both at the Klaipeda site) were noted after discharge. The adverse events included one intraoperative bladder injury (severity:severe, conversion to open), two postoperative haemorrhages (severity: severe and moderate), one postoperative ileus (severity: moderate), one urinary tract infection (severity:mild), and one pelvic peritonitis (severity:moderate). All adverse events were defined as unrelated to the Senhance Surgical System except for the bladder injury. The causality of the bladder injury to the Senhance Surgical System was assessed as probable. All adverse events were effectively treated with appropriate interventions, and patients were discharged in good health. No death in relation to the procedure or robotic system was reported.

### Pain

Overall pain scores revealed a mild pain level of 2.7 (± 1.2) on a 0–10 visual pain scale one day postoperative. The pain score was successfully reduced at discharge to 0.9 (± 0.5) on average. Results per site can be found in Table 4.

**Table 4:** Pain perception

	N	Klaipeda	Feldkirch	Total
		236	59	295
One day postoperative	Mean ± SD	2.8 ± 0.9	2.1 ± 2.0	2.7 ± 1.2
At discharge	Mean ± SD	1.0 ± 0.3	0.4 ± 0.7	0.9 ± 0.5

## Discussion

The present findings highlight the first multi-site study that examines robotic TH with the help of the Senhance Surgical System. In light of the results of the present study, we can underline various insightful findings.

To get a deeper understanding of our current results, we can contextualise them via a comparison with (a) our previous findings, (b) other studies involving the Senhance Surgical System, and c) outcomes reported for other (robotic) systems and approaches. The duration of surgery in our recent study was recorded at 95.0 min, with an IQR spanning from 85 to 118.5 min. In our earlier publication on the first 100 Gynaecological procedures in Klaipeda [12], we reported an average surgical time of 99 min, ranging from 30 to 185 min, with a standard deviation of 33 min. Notably, most of these cases (81 out of 100) were robotic TH, and our findings indicate a slightly reduced surgery duration trend. Another study [6] found significant differences in surgical times for TH with the Senhance Surgical System (*n* = 26) compared to laparoscopic TH ( *n* = 34), with a median of 138.5 min versus 97.5 min, respectively (*p*-value < 0.001). Our study displays a sample size approximately ten times as large and underlines that with larger case numbers, routine settles in, and times can be achieved similarly and even below laparoscopic surgery. We also provided an example illustrating active console time from Feldkirch. Of the reported 95 min, 68 min (72%) represent active engagement with the robotic system, while the remaining time is allocated, for example, to closing sutures.

Further, our results must be understood in a surgical context. Even though we found a significant difference between sites for docking time (3 min in Klaipeda and 4 min in Feldkirch), variation seems minimal for the procedural technique. For instance, Gueli Alletti et al. [10] reported a median docking time with the Senhance Surgical System of 10.5 min (range: 5–25 min) for a specific cohort of 10 obese women. McCarus et al. [14] performed 15 procedures for which an average docking time of 9.2 min was noted, and Fanfani et al. [8] reported an average docking time of 7 min (range: 3–36) in 146 patients. Regarding all studies, we can find a steady decline in docking time with increasing patient numbers, and a routine in the docking procedure is assumable. Importantly, scrubbed nurses/nurses in the OR often perform the docking, so the surgical constellation is not crucial.

An ever-feared event during surgery is the necessity to change the approach, such as transitioning to laparoscopic or open surgery. These scenarios are often critical, requiring a fast transition to prevent subsequent adverse events. Here, the Senhance Surgical System offers a time-saving advantage through its trocar placement in a standard laparoscopic position and trocar sizes compatible with the direct use of laparoscopic instruments. Additionally, the haptic feedback and intuitively self-controlled camera may be relevant. The extra “sensation” and visual overview can prevent the surgeon from applying excessive force or losing focus in a chaotic surgical field. In our previous report [12], we counted six conversions, displaying a conversion rate of 6%. Similarly, Fanfani et al. [7] had to convert approaches in 6.2% (5 cases). A large study of 1051 laparoscopic TH [15] found a conversation rate of 5.0% to open surgery. Guelli Alletti et al. (Gueli Alletti et al. 2018) recorded no conversion to laparoscopic surgery, but their case size was as small as ten patients. Our current findings (three conversations, 1%) are minimal and suggest good control within the operating field and, thus, feasibility and safety with the robotic system.

Equipment malfunction and limitations are other concerns in minimally invasive surgery, particularly robotic surgery. This concern is rational, given that the surgeon and assistant do not directly control the manoeuvring directly but depend on a third medium. The robotic limitations in our study highlighted a notable prevalence of limited motion and collision events, which warrants a closer examination of the specific details. Limited motion, in particular, emerged as a critical finding. During surgery, when the Senhance Surgical System detects potentially limited motion, it issues a warning signal to the surgeon's display. It is crucial to understand that with sufficient experience, this anatomically challenging situation can be delicately manoeuvred and managed. Hence, experienced surgeons could successfully navigate through such situations. Further, the aspect of collision is particularly intriguing. Collision is mainly associated with limited space in the operation room (OR), especially if four arms are used. Hence, it is important to note that our study sites differed in how their operating rooms were utilised. In Feldkirch, the OR is exclusively used for gynaecological procedures, optimising the space for these specific surgeries. Conversely, in Klaipeda, the OR is utilised for various procedures from other departments, potentially resulting in less optimal conditions for the Senhance Surgical System and its arms. This robotic limitation was most often promptly solved via manual adjustments at the table site. Notably, robotic malfunctions were only found in a minority of cases, with no significant differences between sites.

The most significant finding of the present study pertains to adverse events. Here, only a minimal number of six events (2%) were documented, and all events were directly linked and typical for this specific procedure, suggesting no exceptional event. To emphasise, only one occurred intraoperatively. This outcome aligns with or even falls below what is reported in the existing literature. McCarus [14] found three adverse events in 15 cases. They were described as: “postoperative vaginal bleeding”, “gas pain”, and “gas pain with constipation” [14]. A comprehensive study by Rosero et al. (Rosero et al. 2013) revealed no significant difference in adverse events between robotic TH (4.78%) and laparoscopic TH (4.35%, *p*-value = 0.205). Notably, the robotic system was not explicitly specified, and data was collected from 7.788 closely matched patients in each cohort.

Subjective pain perception is crucial in surgery and significantly influences patient outcomes. Minimising pain enhances the overall patient experience and contributes to a faster recovery, diminished stress response, and shorter hospitalisation. Our findings indicate that pain was already minimal postoperative and was successfully further reduced to a minimum level at discharge. This is consistent with pain management goals observed in laparoscopic surgery, emphasising the importance of effective pain control for improved surgical outcomes [16].

In light of ongoing advancements, optimising robotic systems warrants further attention. While 5 mm instruments stand as the norm in robotic surgery, laparoscopic surgery obtains a broader variety of tools, including smaller sizes. The Senhance Surgical System distinguishes itself as one of few robotic systems offering 3 mm instruments, ultrasonic devices, and articulating instruments. Notably, Montlouis-Calixte et al. [17] reported complete safety in their initial experience with 3 mm instruments in 14 patients, of whom 9 underwent gynaecological surgery. The next step might be integrating augmented reality, representing a significant stride toward an enhanced level of safety.

### Limitations

Our study has several limitations. Firstly, we did not analyse other techniques (e.g., laparoscopic or open surgery) in direct comparison. Secondly, we did not include long-term outcomes or participant follow-ups. Thirdly, the patient selection at both sites was likely to be guided by selecting “robotic” feasible patients.

## Conclusion

Based on the results of 295 robotic-assisted TH, it has been demonstrated that many cases contribute to a well-established routine with the applied procedure and system. Procedure times were recorded as minimal and notably, adverse events and robotic malfunctions were infrequent, indicating good control. The Senhance Surgical System proves to be both feasible and safe in the context of total hysterectomies.

## Data Availability

Not applicable.
